# Perceptions of the roles, impact, challenges and needs of community drug distributors in the control and elimination of neglected tropical diseases in difficult-to-access communities in Ghana

**DOI:** 10.1186/s12879-023-08437-z

**Published:** 2023-07-10

**Authors:** Akua Obeng Forson, Raphael Baffour Awuah, Abdul Rahim Mohammed, Christopher Mfum Owusu-Asenso, Sefa Bonsu Atakora, Gabriel Akosah-Brempong, Anisa Abdulai, Isaac Kwame Sraku, Shittu B. Dhikrullahi, Simon K. Attah, Yaw Asare Afrane

**Affiliations:** 1 Centre for Vector-borne Diseases Research, Department of Medical Microbiology, Medical School, University of Ghana, University of Ghana, Korle-Bu, Accra, Ghana; 2 Department of Medical Laboratory Science, School of Biomedical and Allied Health Sciences, University of Ghana, Korle-Bu, Accra, Ghana; 3 Regional Institute for Population Studies (RIPS), University of Ghana, Legon, Accra, Ghana

**Keywords:** Community drug distributors (CDDs), Neglected Tropical Diseases (NTDs), Community’s perceptions, Challenges, Ghana

## Abstract

**Introduction:**

The success of mass drug administration (MDA) campaigns to control and eliminate neglected tropical diseases (NTDs) in Ghana depends, to a large extent, on the essential role community drug distributors (CDDs) play. This study aimed to investigate community’s perceptions of CDDs’ roles, impact of CDDs’ work, challenges faced by CDDs, and views on resources required to enhance CDDs’ work to sustain MDA campaigns.

**Methods:**

A cross-sectional qualitative study employing the use of focus group discussions (FGDs) with community members and CDDs in selected NTD endemic communities together with individual interviews with district health officers (DHOs) was conducted. We interviewed 104 people aged 18 and over, purposively selected, through eight individual interviews, and 16 focus group discussions.

**Results:**

Participants in the community FGDs noted that health education and the distribution of drugs were the main roles of CDDs. Participants also perceived that the work of CDDs had prevented the onset of NTDs, treated symptoms of NTDs, and generally reduced the incidence of infections. In the interviews with CDDs and DHOs, lack of cooperation/non-compliance by community members, demands by community members, lack of working resources and low financial motivation were mentioned as the main challenges to the work of CDDs. Moreover, the provision of logistics and financial motivation for CDDs were identified as factors that will enhance their work.

**Conclusions:**

Incorporating more attractive schemes will incentivise CDDs to improve output. Addressing the challenges highlighted is an important step for the work of CDDS to be effective in controlling NTDs in difficult-to-access communities in Ghana.

## Introduction

Neglected Tropical Diseases (NTDs) are a diverse group of infections and conditions that thrive in tropical and subtropical countries, and cause significant morbidity, social stigma, and death [1–8]. It is estimated that more than one billion people in the world are affected by one or more NTDs [9]. In Ghana, more than three hundred thousand people are affected by lymphatic filariasis, onchocerciasis, schistosomiasis and soil-transmitted helminthiases (hookworm infection, ascariasis, and trichuriasis) [10]. For most of these infections, preventive chemotherapy (PC) is effective at reducing transmission and prevalence [11]. However, for NTD control programmes to be successful, preventive chemotherapy must be given annually or bi-annually to an entire population at risk [mass drug administration (MDA)] or specific at-risk groups (targeted treatment) to prevent new morbidity developing whilst treating individuals with the infection to reduce or interrupt disease transmission [12].

In Ghana, the main strategy to control NTDs is mass drug administration, and community drug distributors are mostly used to deliver drugs to whole communities as part of the control of NTDs [13–15]. Community drug distributors (CDDs), are usually volunteer members of the disease-affected community, recruited to distribute drugs within their resident communities [13]. CDDs often develop a good rapport with community members, and ensure they take their drugs [14]. A review by Krentel et al. [16]. reported on the role that CDDs play, both as positive and negative influences on MDAs. Some of the positive ways that CDDs impacted coverage and compliance included: visiting the household prior to MDA; providing motivation by directly taking the drugs in front of community members and continuous interaction with the community members prior and post treatment of MDA [16]. CDDs can also negatively impact MDA activities when there is low motivation from inadequate supervision from health staff, inadequate training and incentives, and delays in drug supplies [17]. Community perceptions of CDDs are also crucial in influencing uptake of NTD activities. In a study on onchocerciasis control in Southwestern Ethiopia, individuals who perceived CDDs as being properly trained on the diseases, and the treatments were more likely to comply with the treatment than individuals who perceived CDDs were poorly trained [18]. A study in Kenya on schistosomiasis revealed that community members’ perceptions about relations between CDDs, and targeted groups as well as conspiracy theories about the disease not being life-threatening were likely to refuse or adhere to treatment [19]. However, in Uganda, increased perceptions of personal risk of onchocerciasis infection, (advocated by CDDs), was associated with high treatment compliance [20].

In Ghana, the sustainability of MDA campaigns for NTDs in difficult-to-access communities is of concern mainly because of a lack of clarity in the responsibilities of CDDs under contemporary MDA procedures, which require them to deliver multiple drug combinations and maintain different reporting forms [13]. Furthermore, anecdotal reports suggest that CDDs in many communities have either dropped out as volunteers or are not executing their roles as expected, mainly because of resource constraints to execute MDA campaigns.

This study sought to assess community perceptions of CDDs’ roles, the impact of their work, challenges faced, and needs required to enable them to work effectively to sustain MDA campaigns in difficult-to-access areas in Ghana. It also identifies some of the implementation challenges and opportunities to improve MDA uptake from the perspectives and experiences of the CDDs and district health officers. A previous study in Northern Ghana assessed the level of lymphatic filariasis MDA coverage and explored factors affecting its implementation [21]. This present study goes further to explore the challenges in the implementation of MDA and the work of CDDs in difficult-to-reach areas, which is important because MDA seems to be neglected in such areas. It is expected that this assessment will provide useful insights into understanding the contribution, peculiar challenges, and needs of CDDs towards the control and elimination of NTDs in Ghana.

## Methods

### Study sites

**Fig. 1:**
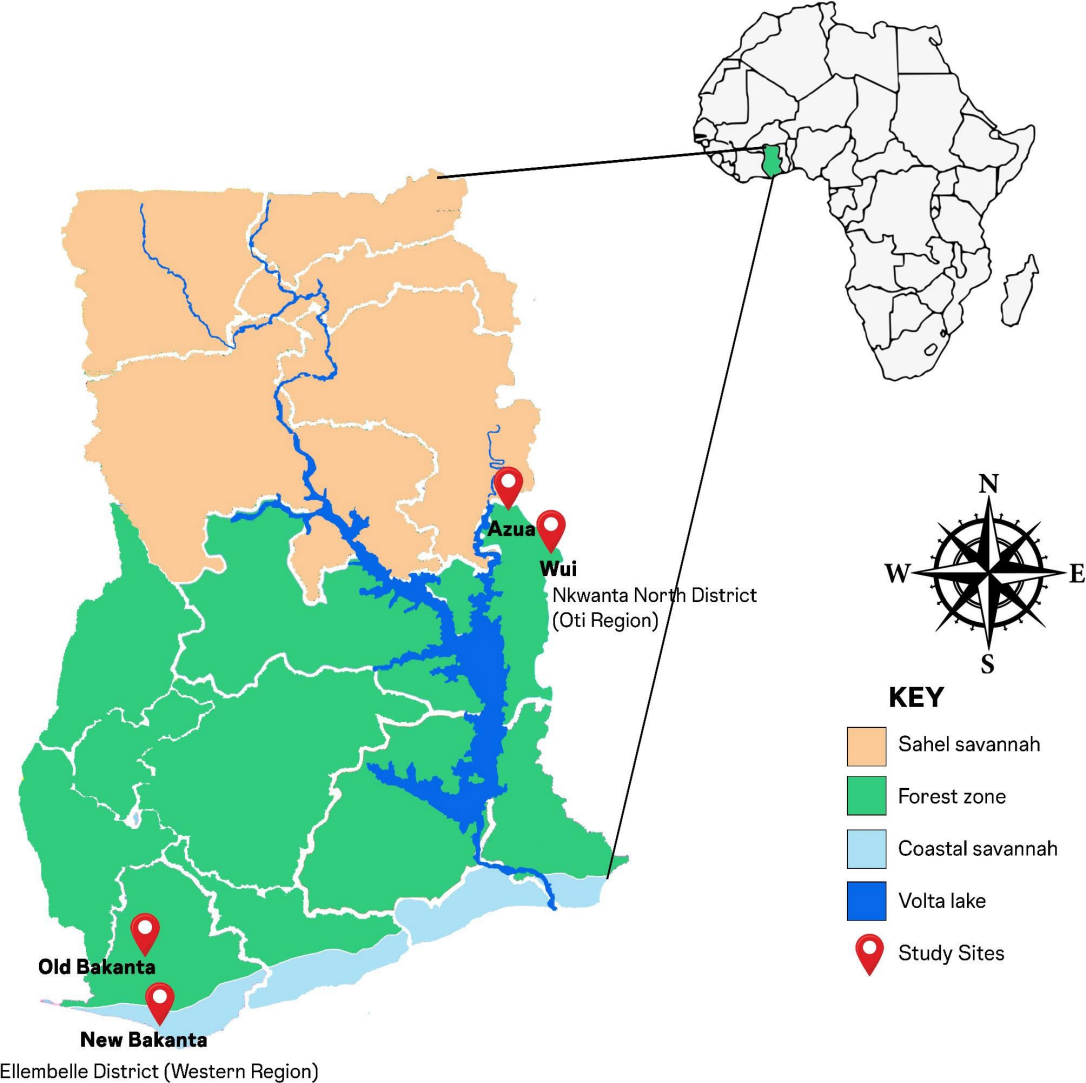
Map showing the study sites used in this study

This was a cross-sectional qualitative study carried out in: Old Bakanta and New Bakanta in the Ellembelle district of the Western region, and Azua and Wui in the Nkwanta North district of the Oti Region (Fig. 1). These communities were targeted because they are difficult-to-access (border towns with limited road network access and some accessible by paddled canoes), endemic to lymphatic filariasis (LF), and onchocerciasis. Since 2012, the study sites have benefited from MDA twice a year, with recent MDA occurring in the sites from January to June 2018 prior to this study. Based on the heights of the participants, they are given ivermectin (1 – 4 tablets) and a tablet of albendazole for lymphatic filariasis, and for onchocerciasis, ivermectin is administered. The residents of these communities lack proximal access to health care for LF, onchocerciasis and other communicable/infectious diseases.

### Population studied, samples

The study employed the use of focus group discussions (FGDs) with community members and CDDs, and individual interviews (IIs) with district health officers (DHOs) from the selected NTD endemic communities described above. Criteria for selection of participants were for members of the community to be aged at least 18 and over and having lived at least 5 years in the study sites. Participants were selected using a purposive sampling strategy. Participants were contacted by community volunteers and research team members, and once the study was explained to them and consent given, they were invited to participate in the study. In each community, four FGDs were conducted – two with younger adults (18–35 years) and another two with older adults (36 years and older). In addition, one FGD was conducted with CDDs in each community. The community FGDs had between 8 and 10 participants while the FGDs with CDDs had an average of 5 participants. Furthermore, individual interviews were conducted with the DHO in each district.

### Collection of data

Data was collected from the FGDs involving community members and CDDs, as well as from the individual interviews with the district health officers (DHOs) during November, 2018 and December, 2019. The FGDs aimed to explore the community’s knowledge and perceptions of CDDs and their role in NTDs lymphatic filariasis and onchocerciasis control activities. FGDs and individual interviews (IIs) were also conducted with CDDs and district health officers (DHOs) respectively to assess challenges faced by CDDs and suggest tools/resources required to enhance their work to sustain MDA campaigns.

Participants in both IIs and FGDs, had to answer questions based on an interview guide. FGDs were conducted in community settings such as schools, churches, and quiet places chosen by community members. The average duration of the FGDs was one hour. Individual interviews were conducted at locations convenient to and chosen by the informant. The focus group recordings and interviews (retrieved from field notes and audio recordings taken by the members of the research team) were transcribed verbatim from the local language (Nzema and Kokomba) into English, respecting idioms. These interviews lasted an average of 45 min to an hour.

### Themes

The organizing themes from the analysis of the FGDs and IIs were centered around the obejctives of the study. These were, perceptions of CDDs’ roles, perceptions of impact of CDDs, challeneges faced by CDDs, and requisite resources deemed to enhance work of CDDs. The first organizing themes reflects the analysis of the FGDs with community members. The second theme reflects analysis of the FGDs and IIs with CDDs and DHOs respectivley whilst the third reflects analysis of all categories of participants. The organizng themes and associated codes are described below.

### Ethical considerations

Ethical clearance was sought from the Institutional Review Board, College of Health Sciences, University of Ghana (Ethical approval no: CHS-ET/M.2-4.10/2018–2019). Informed consent was sought from participants before involvement in the study.

### Data management and analysis

FGDs and IIs were audio recorded with the permission of respondents, and saved on a password-protected computer. The data were transcribed by trained personnel with prior experience in the transcription of recorded qualitative interviews.

Transcripts were analysed using the thematic analytical approach described by Attride-Stirling [22]. Quotes that best capture the essence of what was said by participants in local languages, during the interviews, were appropriately represented for illustration. Analysis of the data was facilitated by Atlas.ti (version 7.5).

## Results

Table 1 shows the socio-demographic characteristics of the study participants. A majority of the study participants were male, had some education, and were over 35 years of age. The following results represent a summary of the opinions expressed by the participants and are illustrated by quotes from the transcript of both individual and group interviews.

**Table 1:** Selected socio-demographic characteristics of FGD participants

Characteristic	No. (%)
**Sex**	
Male	68 (65)
Female	36 (35)
**Education**	
No education	36 (35)
Primary	24 (23)
JHS	20 (19)
SHS	16 (15)
Tertiary	8 (8)
**Age group**	
18–35	36 (35)
36 and over	68 (65)

## Perceptions of CDD’s roles

In all the FGDs with community members, it was noted that the main roles of the CDD are to provide health education and distribute drugs. In two of the FGDs for example, participants indicated how CDDs are expected to be sources of health knowledge.

“They (CDDs) are supposed to educate us on protecting ourselves from the disease and how to treat the disease when we are affected” *[FGD with younger adults in an onchocerciasis endemic community].*

“The community ‘drug people’ are to give us education on disease prevention. They should be equipped with the relevant knowledge all the time to give us important information that will guide us to prevent diseases and the advice on medicines to take when we are not well” *[FGD with younger adults in an LF endemic community].*

In another FGD, participants pointed out that CDDs are expected to inform community members when drugs are available and when distribution will be done.

“They should give announcements when the drugs have arrived and that they will start distributing in the morning or in the evening” *[FGD with older adults in an onchocerciasis endemic community].*

### Perceptions of impact of CDDs

Community members expressed their perceptions on the impact of the work of CDDs. Participants in three of the FGDs perceived a positive impact of the work of CDDs on NTD control. In one of the FGDs with younger adults, participants pointed out that the work of CDDs, through the MDA, reduced the incidence of infections.

“I can say from what I have observed that the rate of infections of the disease (LF) in the community has reduced compared to 10 years ago because of the work of the community drug volunteers, through the drugs they have been giving us when they come into our homes” *[FGD with younger adults in an LF endemic community]*

Participants in the community FGDs also perceived that the work of CDDs have facilitated the MDA and individuals in the community with symptoms of NTDs have been treated or cured. Some participants in both the young and older adults FGDs indicated thus:

“You may now be developing symptoms of the disease (LF), but when you take the drug given during the distribution, it will cure it completely. So, the drug distribution has been very helpful in curing people who have shown symptoms of infections” *[FGD with younger adults in an LF endemic community].*

*“*If you become infected and you continue to take the drug which is brought to us by the community drug distributors, you will be cured” *[FGD with older adults in an LF endemic community].*

In another group discussion, participants mentioned that the efforts of CDDs have helped to prevent the onset of NTDs, particularly, onchocerciasis. The following was noted in one of the FGDs with older adults:

“The benefits from the drugs given to us by the community drug distributor is that, if you are to continue taking the drug, it will prevent the disease (onchocerciasis) in the community” *[FGD with older adults in an onchocerciasis endemic community].*

However, a few FGD participants pointed out some negative aspects of the work of CDDs. In one of the older adults FGDs, participants noted that the untimely delivery of drugs by CDDs was negatively impacting the MDA.

“When they start distributing the drug, they come to your house, measure your height and give you the drug. That is what they do. Sometimes they don’t come at a good time. When they come to meet your absence, they will have to go and they may not come back. If this happens, we can’t see any benefit from their work” *[FGD with older adults in an onchocerciasis endemic community].*

Participants in one of the older adults’ groups also indicated that the perceived side effects of the drugs taken during MDA visits is sometimes not fully explained by the CDDs. Some participants in the older adults FGDs had these to say:

“A woman was complaining of waist pains and body pains after she took the drugs…the second time the drugs were distributed; a boy was complaining of chest pains, so, he was sent to the hospital. The CDDs should have told us of these side effects” *[FGD with older adults in an LF endemic community].*

“Some people develop skin rashes and others have spots on their skin when they take the drugs but all these have not been explained to us by the people who give us the drugs. Because of this, I don’t see the importance of their work if they can’t explain this to us” *[FGD with older adults in an LF endemic community].*

In addition, FGD participants in the LF endemic communities noted that MDA visits by CDDs occurred at least once a year. A participant in one of the FGDs said this:

“It’s more than 6 months because the last time they distributed the drugs was about a year ago. I don’t know if they have done another mass distribution, but I know it’s been a year since they distributed it here. We cannot benefit from this drug distribution if it continues this way” *[FGD with younger adults in an LF endemic community].*

### Challenges faced by CDDs

Challenges faced by CDDs in the implementation of MDA were highlighted in the FGDs with CDDs and interviews with DHOs. The major constraints to the work of CDDs mentioned were the lack of cooperation/ non-compliance by community members, demands by community members, lack of working resources and low financial motivation.

CDDs noted that community members sometimes do not cooperate with them or adhere to their advice to take the drugs in their presence for a number of reasons, including the possible experience of side effects. A participant in one of the group discussions with CDDs noted this:

“Sometimes we visit households and there is someone who has the disease (onchocerciasis), but he/she is not ready to take the drug. At that point you have to educate the person on the importance of the drug so that the person takes it. We are not given the drugs to keep but to distribute and make sure it is taken before we leave…because of the side effects they experienced in the beginning, some people don’t want to take the drugs” *[FGD with CDDs in a onchocerciasis endemic community].*

In addition, CDDs highlighted that there had been times where some community members demanded to be given food and/or money before cooperating with them. A participant in one of the group discussions with CDDs had this to say:

*“*Some people demand food and money when we go distributing the drugs, and this sometimes is a problem” *[FGD with CDDs in an LF endemic community]*

The lack of working accessories/resources to enable CDDs work efficiently was mentioned as a challenge in one of the interviews with DHOs. It was noted that even though some CDDs have some basic working resources, they lacked several others.

“Sometimes they will go meet someone’s absence and they will have to go there again, probably in the evening. A proposal was written and they were given bicycles, but they don’t even have raincoats or rubber boots during the raining season” *[Interview with a DHO]*

One of the CDDs also said this in relation to the lack of working accessories/resources.

When you have raincoats and you are distributing drugs, the drugs won’t get wet when it rains. We lack rubber boots and there are snakes in this community which can bite you at night” *[FGD with CDDs in an onchocerciasis endemic community]*

In another group discussion with CDDs, it emerged that the lack of financial motivation was a challenge to the effectiveness of their work given the demands and dangers associated with their role.

“Our challenge is financial. Sometimes the money we get from training is Ghc40 (≈$7) and the money we get from attending a workshop is Ghc60 (≈$11). When you are bitten by a snake at night as you undertake your duties, the Ghc60 (≈$11) cannot take care of you” *[FGD with CDDs in an onchocerciasis endemic community]*

### Main resources needed by CDDs to enhance their work

Community members, CDDs and DHOs further expressed opinions on how to enhance the work of CDDs to sustain the MDA campaigns to control NTDs. Provision of logistics to enable CDDs undertake regular MDA visits, financial motivation and cooperation by community members were mentioned as the main resources needed by CDDs.

In one of the group discussions with CDDs, a participant said this:

“If we want to improve the prevention of this disease then we should be given the necessary logistics or resources (materials for disseminating information, recording and reporting information associated with drug distribution and adverse events) so that we can give the drugs consistently and expand our reach” *[FGD with CDDs in an LF endemic community]*

Similarly, one of the DHOs mentioned that:

“Equipping CDDs with the basic needs (branded T-shirts, bags, hats, boots rain coat and rubber boots) will facilitate their work and ensure the effectiveness of the MDA” *[Interview with DHO]*

In the community FGDs, participants stated that CDDs should be financially motivated to ensure the sustainability of the MDA.

*“*Like we are saying, they (CDDs) work but they don’t have money, so if they will get some money it will enable them to work well so the disease is also contained. They have to be given money to motivate them” *[FGD, LF prevalent community, older adults]*

Contrary to assertions for CDDs to be provided with the specific needs mentioned above, some participants in the community FGDs noted CDDs rather need to be tolerant with community members to ensure sustainability and effectiveness of the MDA.

“They have to explain and be tolerant with people to take the drug. Someone might not know why they should take the drug, so they have to explain things to them” *[FGD with younger and older adults in an LF endemic community].*

## Discussion

This study explored the perceptions of CDD roles by community members, impact, challenges faced and views on resources needed by CDDs to enable them work effectively on the delivery of a community-wide MDA program for the control and elimination of NTDs in Ghana.

Adequate health education enables community members to appreciate the fact that NTDs are conditions that could bring devastating outcomes. The FGDs with the community members revealed that they perceived that the incidence of infections had reduced due to the MDA and indicated that people with symptoms of NTDs have also been cured. These findings align with the assertion that MDA accelerates elimination of NTDs by reducing the number of infections [23]. These perceived positive impacts of MDA activities have previously been reported by community members in Indonesia [24] and Tanzania [25]. In addition, community members appreciated the distribution of drugs by the CDDs, but wished to be better informed when drugs are available for distribution. Community members also had positive impressions on the impact of the MDA.

The FGDs with the community members revealed that CDDs were sometimes unable to adequately discuss and give health advice while distributing the drugs. Such poor interaction and communication gaps can constitute a huge implementation barrier. Studies from Kenya [26] and Ethiopia [18] have shown that gaining community acceptance, trust and connectedness through health education of the community members by the CDDs is a deciding factor on whether or not community members would receive them, and accept the medicines. In some districts in India, the training of CDDs with the necessary knowledge, and skills to conduct health education positively improved the compliance rate [27]. There is a need to invest in continuous training and education of CDDs to shore up their tolerance and confidence while dealing with, and answering questions posed by community members during MDA.

Despite an overall awareness and knowledge of the presences of CDDs in the communities, several issues arose that place an emphasis on the need for increased sensitisation and education of community members. In the community FGDs, participants mentioned that untimely drug delivery was a major challenge they have with CDDs. This finding contradicts that of a study in Kenya which reported that timely drug delivery was one of the best practices by CDDs in ensuring prompt uptake of drugs [28]. The difference in results could be due to logistical and behavioural differences in the different countries. Hodges et al., [29] study in Sierra Leone on MDA of lymphatic filariasis has reemphasized the positive implications for community participation in MDA when an appropriate drug delivery strategy is instituted to reach all community members. The current study recommends the NTDs program should consider different delivery approaches so as to reach all infected members in endemic communities.

Nonetheless, participants in the community FGDs mentioned that the perceived side effects of the drugs, such as swollen legs, body/chest pains, skin rashes/spots and diarrhoea were a major challenge to the success of the MDA. One key enabler to compliance of MDA in endemic communities is to alleviate fears by properly educating participants on the possible occurrence of adverse reactions. Studies conducted in Brazil [30], Kenya [31], India [32, 33], and Haiti [34] have shown that fear of adverse reactions was the main reason for systematic noncompliance in the populations they studied. Non-compliance with treatment can be a serious obstacle to the control and elimination of NTDs. A study by Babu et al., [27] revealed that communities with stationed medical officers and health workers to attend to community members with adverse side effects, at local primary health centres, witnessed highly successful MDA programmes. Hence, the success of NTDs elimination largely depends on designing an MDA programme where the Ghana Health Service put in measures to promptly reach out to participants that experience severe reactions after taking the drugs. Also, measures have to be put in place to address the issue of health facilities demanding for payment before treating those who report at the centres with adverse side effects [35].

Furthermore, FGDs with participants in the LF endemic communities revealed that MDA visits by CDDs was not consistent, with some occurring once in a year. In Ghana, MDA usually takes place 1–2 weeks between March and June at different endemic communities [36]. Even then, these programs could be halted for a number of reasons. However, the decision to stop most MDA is always taken at the district level of the Ghana Health Services (GHS), and this is often not communicated to the community members. Such discontinuation can have adverse effects on endemic communities with low coverage of MDA, high baseline endemicity of microfilaria (mf) and logistical programme challenges.

### Challenges faced by CDDs

Challenges faced by CDDs in the implementation of MDA were highlighted in the FGDs with CDDs and interviews with DHOs. CDDs mentioned lack of cooperation/non-compliance by community members, demands by community members, lack of working resources and low financial motivation as the main challenges. These findings partly corroborate key findings of a literature review which showed a noticeable perceived decline in motivation of some CDDs, and which could potentially negatively impact the success of control programmes [14].

Lack of monetary incentives or out-of-pocket expenses led to low motivation by CDD volunteer workers. One participant lamented that the money he received from training was inadequate to cover medical expenses should he be injured while administering the MDA. Fleming et al., [37] reported similar findings by CDDs in Uganda with volunteering costs increasing the number of drug deliveries. Emukah et al., [38] study with community-directed distributors of ivermectin during an onchocerciasis-control programme in Nigeria have likewise reported lack of financial remuneration was a major challenge. Predominantly, some monetary incentives were needed to either cater for transportation to collect drugs from the health units and for drug distribution, as well as for lunch whilst carrying out NTD Programme activities. In Ghana, CDDs receive an allowance for lunch, and transport only during training/ workshop which can vary between districts but with minimum being US$ 17 for annual NTD Programme activities. This payment is regardless of the number of drug delivery rounds to be made. In Ghana, CDDs are volunteers selected by the community to distribute drugs [39], and to help improve their performance, results-based financing (reward schemes – monetary or material incentives) can be instituted to motivate CDDs for reaching their targets, such as numbers treated or programme coverage attained [40].

When the CDDs were asked what other non-financial reward in the form of provision of working accessories/resources could be used for compensation, some of them intimated that a rain coat and rubber boots would facilitate programme activities. Studies by Fleming et al. [37, 41], and Njomo et al., [31] have also reported that CDDs in Uganda and Kenya required working accessories such as T-shirts, bags, hats, boots and waterproof coats with the programme logo on them. The provision of these basic resources will encourage the drug distributors to carry out their work efficiently and provide feelings of ownership which will consequently play an important role in the success of the NTDs programme.

In addition, CDDs highlighted that there had been times when some community members demanded to be given food and/or money before cooperating with them, an assertion a participant in one of the group discussions with CDDs made, and was corroborated by others. Lack of cooperation and refusal of community members to participate are highlighted as major challenges influencing NTDs programme in Burkina Faso [42]. Sensitization of community members of roles and responsibility of CDDs prior to commencement of MDAs might increase cooperation, as well as appreciation from community members on the volunteer services being rendered by CDDs [26]. Furthermore, the lack of financial (or non-financial) incentives discouraged CDDs from following up on community members who were absent during treatment period (absentees due to travel or relocation). Thus, the provision of in-kind incentives for CDDs who are persistent in follow-ups for those who missed treatment could be considered, alongside stocking community health centres and increasing the awareness of treatment sources outside MDA programme. Such initiatives are envisaged to intensify the commitment of the CDDs to the health initiatives.

### Study limitation

The main limitation of the study is the potential recall bias of some CDDs who distributed drugs during the 2017 and 2016 MDA rounds and were interviewed some months after the campaign.

## Conclusion

Despite the limitation, this study has provided crucial information on community members’ perceptions and critical factors influencing CDDs’ motivation in MDA for NTDs elimination. To sustain the MDA, participants recognized a gap in knowledge of CDDs, leading to vocal requests for better information dissemination of MDA activities and assurances of management of infected participants’ safety and adverse effects. In addition, there is a need to identify effective resources and novel approaches to better equip and facilitate the work of CDDs (including financial motivation) and encourage cooperation by community members (which should be spearheaded by community leaders).

## Data Availability

The datasets used and/or analysed during the current study are available from the corresponding author on reasonable request.
